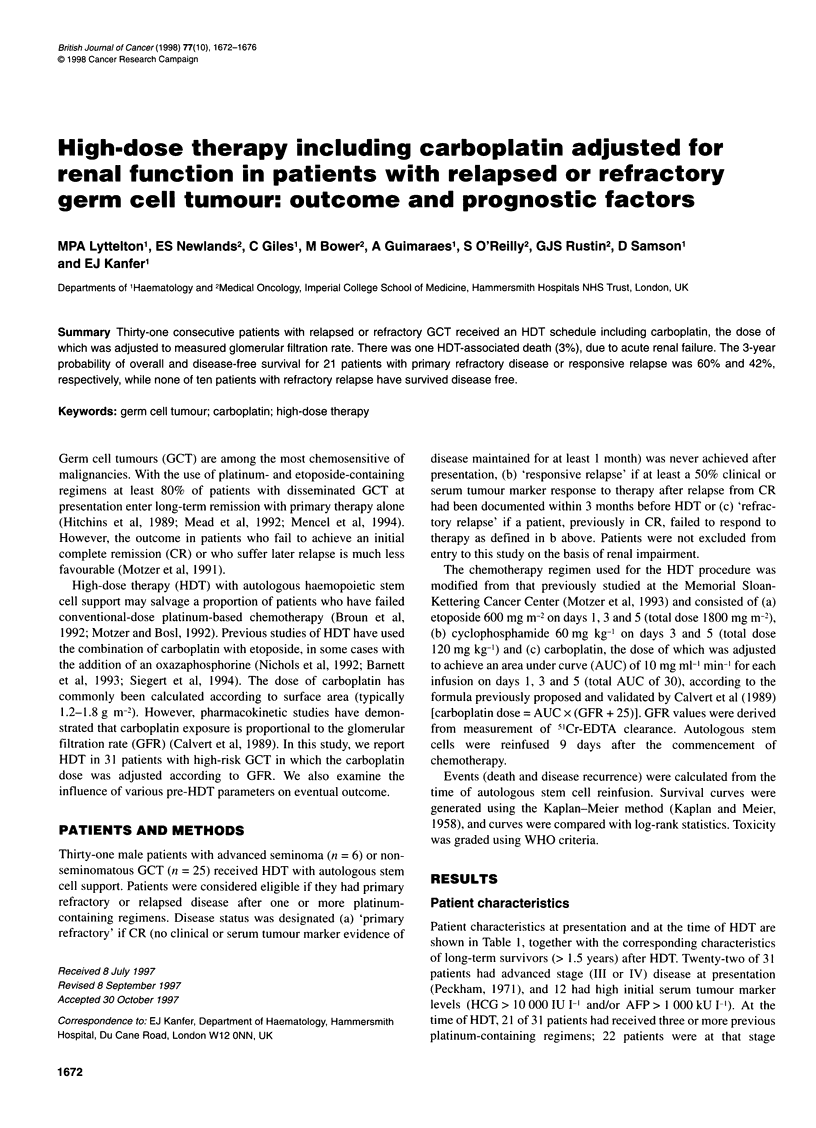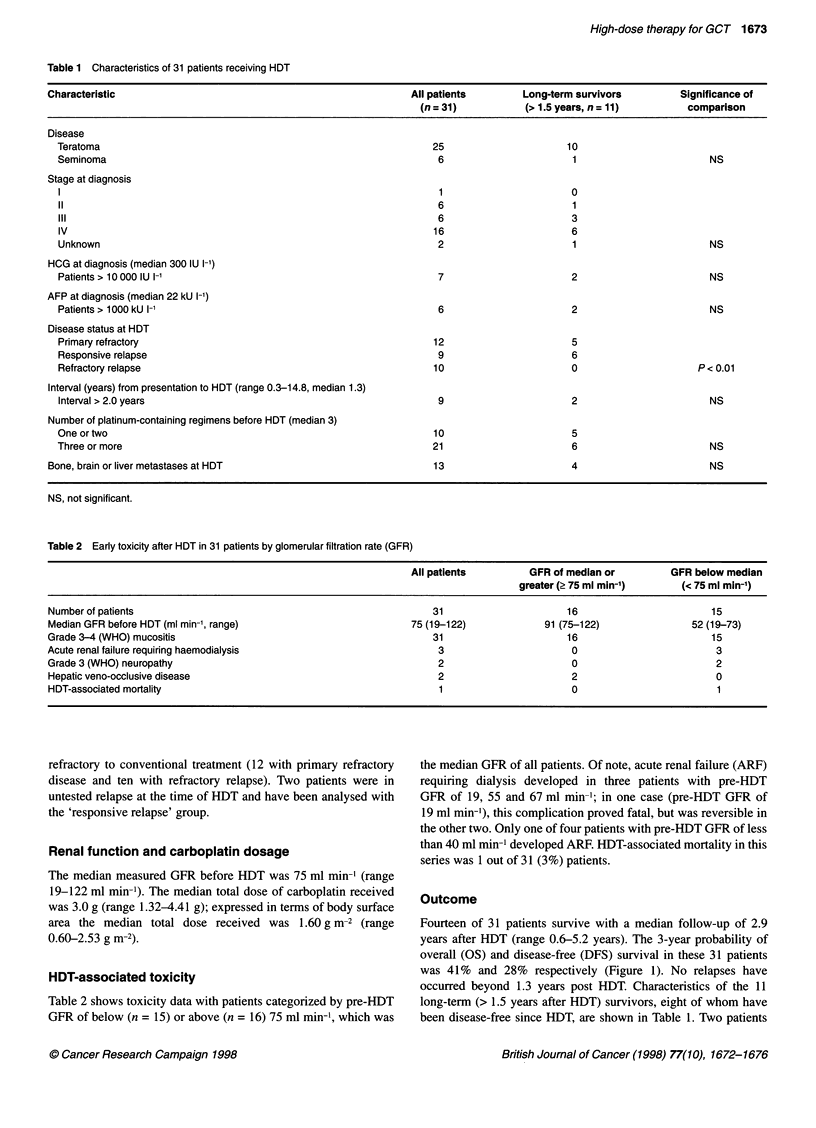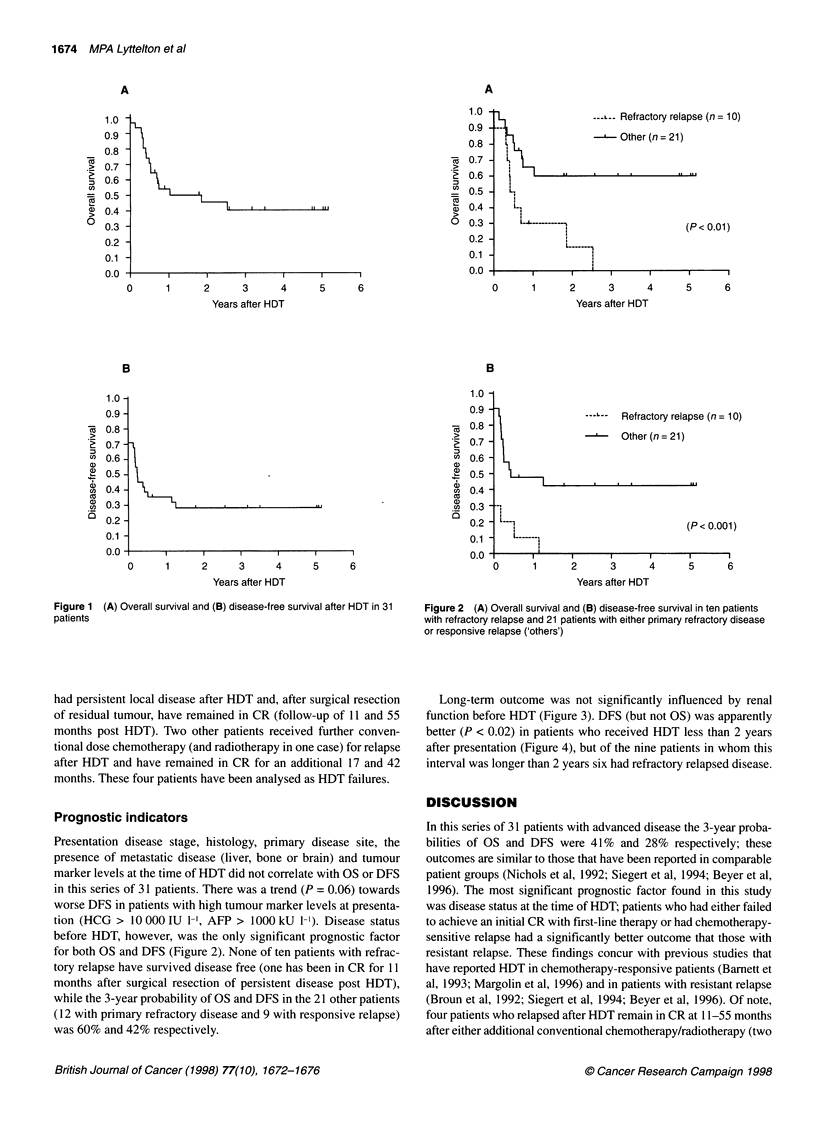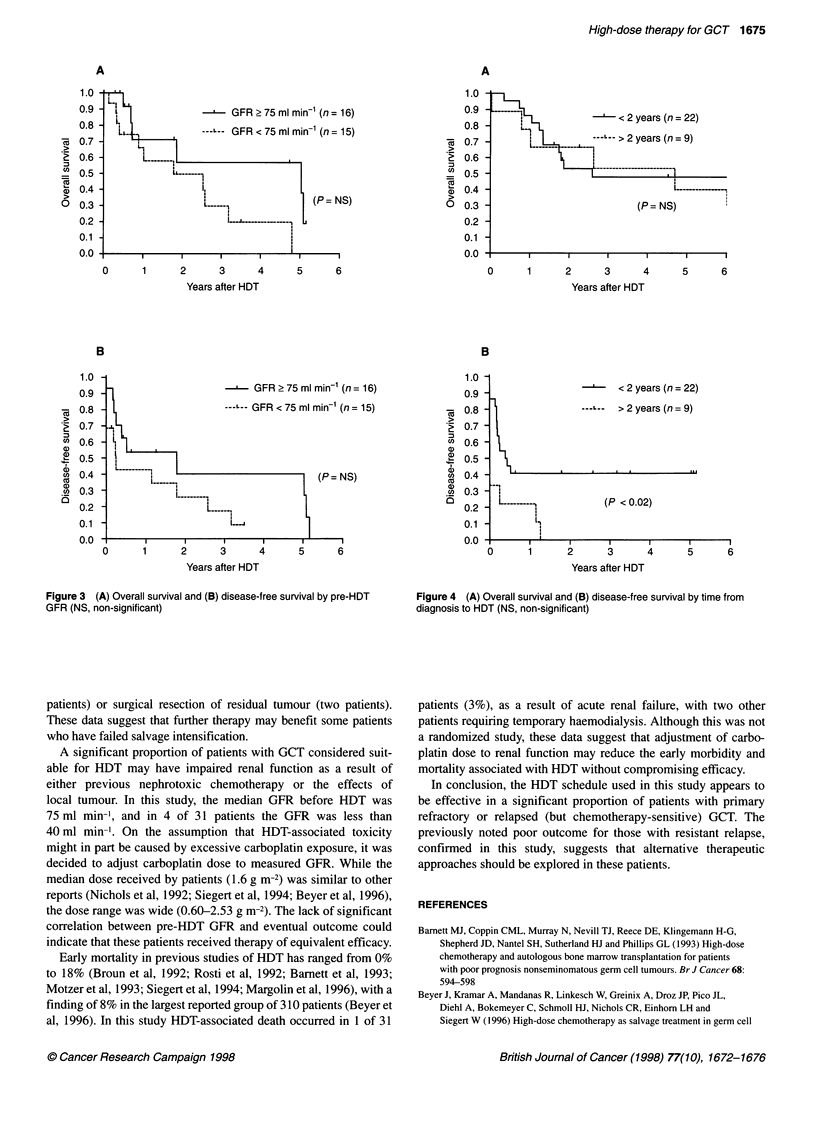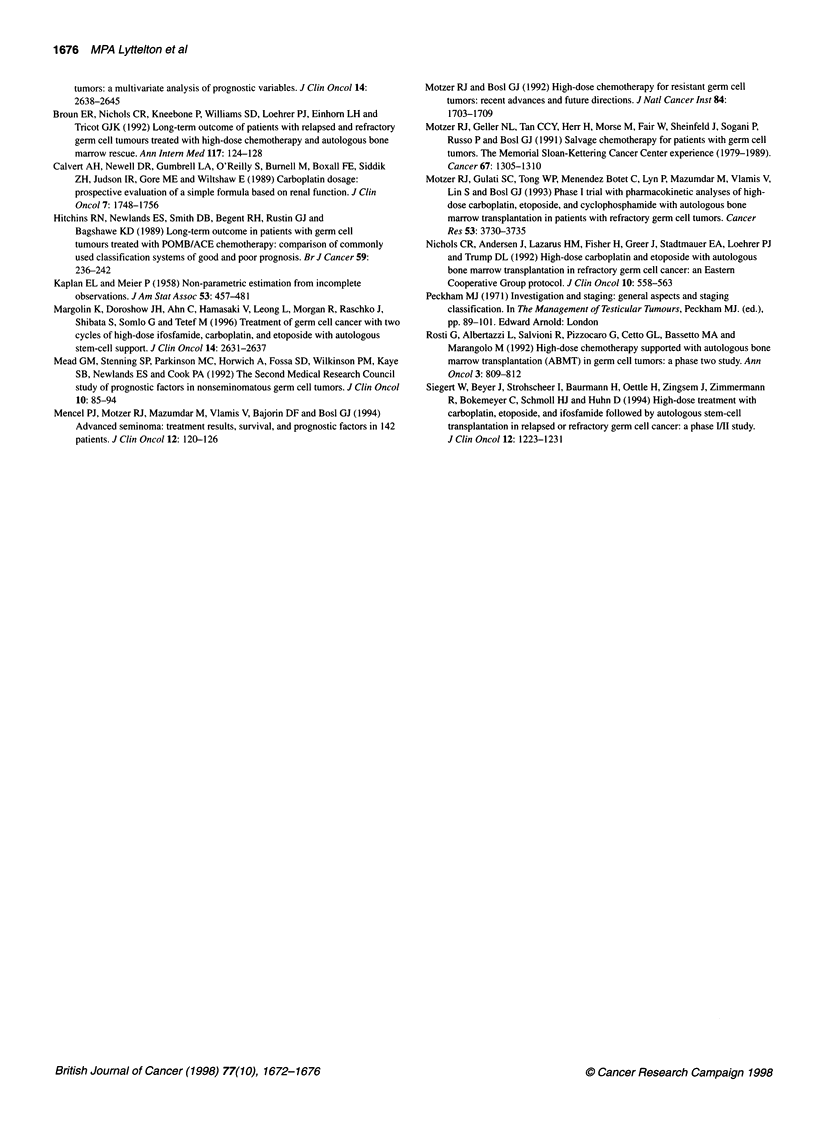# High-dose therapy including carboplatin adjusted for renal function in patients with relapsed or refractory germ cell tumour: outcome and prognostic factors.

**DOI:** 10.1038/bjc.1998.275

**Published:** 1998-05

**Authors:** M. P. Lyttelton, E. S. Newlands, C. Giles, M. Bower, A. Guimaraes, S. O'Reilly, G. J. Rustin, D. Samson, E. J. Kanfer

**Affiliations:** Department of Haematology, Imperial College School of Medicine, Hammersmith Hospitals NHS Trust, London, UK.

## Abstract

Thirty-one consecutive patients with relapsed or refractory GCT received an HDT schedule including carboplatin, the dose of which was adjusted to measured glomerular filtration rate. There was one HDT-associated death (3%), due to acute renal failure. The 3-year probability of overall and disease-free survival for 21 patients with primary refractory disease or responsive relapse was 60% and 42%, respectively, while none of ten patients with refractory relapse have survived disease free.


British Journal of Cancer (1998) 77(10), 1672–1676
© 1998 Cancer Research Campaign

### High-dose therapy including carboplatin adjusted for renal function in patients with relapsed or refractory germ cell tumour: outcome and prognostic factors

**MPA Lyttelton[1], ES Newlands[2], C Giles[1], M Bower[2], A Guimaraes[1], S O'Reilly[2], GJS Rustin[2], D Samson[1] and EJ Kanfer[1]**

Departments of [1]Haematology and [2]Medical Oncology, Imperial College School of Medicine, Hammersmith Hospitals NHS Trust, London, UK

**Summary** Thirty-one consecutive patients with relapsed or refractory GCT received an HDT schedule including carboplatin, the dose of which was adjusted to measured glomerular filtration rate. There was one HDT-associated death (3%), due to acute renal failure. The 3-year probability of overall and disease-free survival for 21 patients with primary refractory disease or responsive relapse was 60% and 42%, respectively, while none of ten patients with refractory relapse have survived disease free.

**Keywords:** germ cell tumour; carboplatin; high-dose therapy

Germ cell tumours (GCT) are among the most chemosensitive of malignancies. With the use of platinum- and etoposide-containing regimens at least 80% of patients with disseminated GCT at presentation enter long-term remission with primary therapy alone (Hitchins et al, 1989; Mead et al, 1992; Mencel et al, 1994). However, the outcome in patients who fail to achieve an initial complete remission (CR) or who suffer later relapse is much less favourable (Motzer et al, 1991).

High-dose therapy (HDT) with autologous haemopoietic stem cell support may salvage a proportion of patients who have failed conventional-dose platinum-based chemotherapy (Broun et al, 1992; Motzer and Bosl, 1992). Previous studies of HDT have used the combination of carboplatin with etoposide, in some cases with the addition of an oxazaphosphorine (Nichols et al, 1992; Barnett et al, 1993; Siegert et al, 1994). The dose of carboplatin has commonly been calculated according to surface area (typically 1.2–1.8 g $m^{-2}$). However, pharmacokinetic studies have demonstrated that carboplatin exposure is proportional to the glomerular filtration rate (GFR) (Calvert et al, 1989). In this study, we report HDT in 31 patients with high-risk GCT in which the carboplatin dose was adjusted according to GFR. We also examine the influence of various pre-HDT parameters on eventual outcome.

### PATIENTS AND METHODS

Thirty-one male patients with advanced seminoma ($n$ = 6) or non-seminomatous GCT ($n$ = 25) received HDT with autologous stem cell support. Patients were considered eligible if they had primary refractory or relapsed disease after one or more platinum-containing regimens. Disease status was designated (a) 'primary refractory' if CR (no clinical or serum tumour marker evidence of disease maintained for at least 1 month) was never achieved after presentation, (b) 'responsive relapse' if at least a 50% clinical or serum tumour marker response to therapy after relapse from CR had been documented within 3 months before HDT or (c) 'refractory relapse' if a patient, previously in CR, failed to respond to therapy as defined in b above. Patients were not excluded from entry to this study on the basis of renal impairment.

The chemotherapy regimen used for the HDT procedure was modified from that previously studied at the Memorial Sloan-Kettering Cancer Center (Motzer et al, 1993) and consisted of (a) etoposide 600 mg $m^{-2}$ on days 1, 3 and 5 (total dose 1800 mg $m^{-2}$), (b) cyclophosphamide 60 mg $kg^{-1}$ on days 3 and 5 (total dose 120 mg $kg^{-1}$) and (c) carboplatin, the dose of which was adjusted to achieve an area under curve (AUC) of 10 mg $ml^{-1}$ $min^{-1}$ for each infusion on days 1, 3 and 5 (total AUC of 30), according to the formula previously proposed and validated by Calvert et al (1989) [carboplatin dose = AUC × (GFR + 25)]. GFR values were derived from measurement of $^{51}$Cr-EDTA clearance. Autologous stem cells were reinfused 9 days after the commencement of chemotherapy.

Events (death and disease recurrence) were calculated from the time of autologous stem cell reinfusion. Survival curves were generated using the Kaplan–Meier method (Kaplan and Meier, 1958), and curves were compared with log-rank statistics. Toxicity was graded using WHO criteria.

### RESULTS

### Patient characteristics

Patient characteristics at presentation and at the time of HDT are shown in Table 1, together with the corresponding characteristics of long-term survivors (> 1.5 years) after HDT. Twenty-two of 31 patients had advanced stage (III or IV) disease at presentation (Peckham, 1971), and 12 had high initial serum tumour marker levels (HCG > 10 000 IU $l^{-1}$ and/or AFP > 1 000 kU $l^{-1}$). At the time of HDT, 21 of 31 patients had received three or more previous platinum-containing regimens; 22 patients were at that stage

*Received 8 July 1997*
*Revised 8 September 1997*
*Accepted 30 October 1997*

*Correspondence to:* EJ Kanfer, Department of Haematology, Hammersmith Hospital, Du Cane Road, London W12 0NN, UK

**Table 1** Characteristics of 31 patients receiving HDT

| Characteristic | All patients ($n$ = 31) | Long-term survivors (> 1.5 years, $n$ = 11) | Significance of comparison |
|---|---|---|---|
| Disease | | | |
| Teratoma | 25 | 10 | |
| Seminoma | 6 | 1 | NS |
| Stage at diagnosis | | | |
| I | 1 | 0 | |
| II | 6 | 1 | |
| III | 6 | 3 | |
| IV | 16 | 6 | |
| Unknown | 2 | 1 | NS |
| HCG at diagnosis (median 300 IU $l^{-1}$) | | | |
| Patients > 10 000 IU $l^{-1}$ | 7 | 2 | NS |
| AFP at diagnosis (median 22 kU $l^{-1}$) | | | |
| Patients > 1000 kU $l^{-1}$ | 6 | 2 | NS |
| Disease status at HDT | | | |
| Primary refractory | 12 | 5 | |
| Responsive relapse | 9 | 6 | |
| Refractory relapse | 10 | 0 | $P < 0.01$ |
| Interval (years) from presentation to HDT (range 0.3–14.8, median 1.3) | | | |
| Interval > 2.0 years | 9 | 2 | NS |
| Number of platinum-containing regimens before HDT (median 3) | | | |
| One or two | 10 | 5 | |
| Three or more | 21 | 6 | NS |
| Bone, brain or liver metastases at HDT | 13 | 4 | NS |

NS, not significant.

**Table 2** Early toxicity after HDT in 31 patients by glomerular filtration rate (GFR)

| | All patients | GFR of median or greater (≥ 75 ml $min^{-1}$) | GFR below median (< 75 ml $min^{-1}$) |
|---|---|---|---|
| Number of patients | 31 | 16 | 15 |
| Median GFR before HDT (ml $min^{-1}$, range) | 75 (19–122) | 91 (75–122) | 52 (19–73) |
| Grade 3–4 (WHO) mucositis | 31 | 16 | 15 |
| Acute renal failure requiring haemodialysis | 3 | 0 | 3 |
| Grade 3 (WHO) neuropathy | 2 | 0 | 2 |
| Hepatic veno-occlusive disease | 2 | 2 | 0 |
| HDT-associated mortality | 1 | 0 | 1 |

refractory to conventional treatment (12 with primary refractory disease and ten with refractory relapse). Two patients were in untested relapse at the time of HDT and have been analysed with the 'responsive relapse' group.

### Renal function and carboplatin dosage

The median measured GFR before HDT was 75 ml $min^{-1}$ (range 19–122 ml $min^{-1}$). The median total dose of carboplatin received was 3.0 g (range 1.32–4.41 g); expressed in terms of body surface area the median total dose received was 1.60 g $m^{-2}$ (range 0.60–2.53 g $m^{-2}$).

### HDT-associated toxicity

Table 2 shows toxicity data with patients categorized by pre-HDT GFR of below ($n$ = 15) or above ($n$ = 16) 75 ml $min^{-1}$, which was the median GFR of all patients. Of note, acute renal failure (ARF) requiring dialysis developed in three patients with pre-HDT GFR of 19, 55 and 67 ml $min^{-1}$; in one case (pre-HDT GFR of 19 ml $min^{-1}$), this complication proved fatal, but was reversible in the other two. Only one of four patients with pre-HDT GFR of less than 40 ml $min^{-1}$ developed ARF. HDT-associated mortality in this series was 1 out of 31 (3%) patients.

### Outcome

Fourteen of 31 patients survive with a median follow-up of 2.9 years after HDT (range 0.6–5.2 years). The 3-year probability of overall (OS) and disease-free (DFS) survival in these 31 patients was 41% and 28% respectively (Figure 1). No relapses have occurred beyond 1.3 years post HDT. Characteristics of the 11 long-term (> 1.5 years after HDT) survivors, eight of whom have been disease-free since HDT, are shown in Table 1. Two patients

© Cancer Research Campaign 1998

Figure 1 (A) Overall survival and (B) disease-free survival after HDT in 31 patients

Figure 2 (A) Overall survival and (B) disease-free survival in ten patients with refractory relapse and 21 patients with either primary refractory disease or responsive relapse ('others')

had persistent local disease after HDT and, after surgical resection of residual tumour, have remained in CR (follow-up of 11 and 55 months post HDT). Two other patients received further conventional dose chemotherapy (and radiotherapy in one case) for relapse after HDT and have remained in CR for an additional 17 and 42 months. These four patients have been analysed as HDT failures.

### Prognostic indicators

Presentation disease stage, histology, primary disease site, the presence of metastatic disease (liver, bone or brain) and tumour marker levels at the time of HDT did not correlate with OS or DFS in this series of 31 patients. There was a trend ($P = 0.06$) towards worse DFS in patients with high tumour marker levels at presentation (HCG > 10 000 IU $l^{-1}$, AFP > 1000 kU $l^{-1}$). Disease status before HDT, however, was the only significant prognostic factor for both OS and DFS (Figure 2). None of ten patients with refractory relapse have survived disease free (one has been in CR for 11 months after surgical resection of persistent disease post HDT), while the 3-year probability of OS and DFS in the 21 other patients (12 with primary refractory disease and 9 with responsive relapse) was 60% and 42% respectively.

Long-term outcome was not significantly influenced by renal function before HDT (Figure 3). DFS (but not OS) was apparently better ($P < 0.02$) in patients who received HDT less than 2 years after presentation (Figure 4), but of the nine patients in whom this interval was longer than 2 years six had refractory relapsed disease.

### DISCUSSION

In this series of 31 patients with advanced disease the 3-year probabilities of OS and DFS were 41% and 28% respectively; these outcomes are similar to those that have been reported in comparable patient groups (Nichols et al, 1992; Siegert et al, 1994; Beyer et al, 1996). The most significant prognostic factor found in this study was disease status at the time of HDT; patients who had either failed to achieve an initial CR with first-line therapy or had chemotherapy-sensitive relapse had a significantly better outcome that those with resistant relapse. These findings concur with previous studies that have reported HDT in chemotherapy-responsive patients (Barnett et al, 1993; Margolin et al, 1996) and in patients with resistant relapse (Broun et al, 1992; Siegert et al, 1994; Beyer et al, 1996). Of note, four patients who relapsed after HDT remain in CR at 11–55 months after either additional conventional chemotherapy/radiotherapy (two

© Cancer Research Campaign 1998

**Figure 3** (**A**) Overall survival and (**B**) disease-free survival by pre-HDT GFR (NS, non-significant)

**Figure 4** (**A**) Overall survival and (**B**) disease-free survival by time from diagnosis to HDT (NS, non-significant)

patients) or surgical resection of residual tumour (two patients). These data suggest that further therapy may benefit some patients who have failed salvage intensification.

A significant proportion of patients with GCT considered suitable for HDT may have impaired renal function as a result of either previous nephrotoxic chemotherapy or the effects of local tumour. In this study, the median GFR before HDT was 75 ml $min^{-1}$, and in 4 of 31 patients the GFR was less than 40 ml $min^{-1}$. On the assumption that HDT-associated toxicity might in part be caused by excessive carboplatin exposure, it was decided to adjust carboplatin dose to measured GFR. While the median dose received by patients (1.6 g $m^{-2}$) was similar to other reports (Nichols et al, 1992; Siegert et al, 1994; Beyer et al, 1996), the dose range was wide (0.60–2.53 g $m^{-2}$). The lack of significant correlation between pre-HDT GFR and eventual outcome could indicate that these patients received therapy of equivalent efficacy.

Early mortality in previous studies of HDT has ranged from 0% to 18% (Broun et al, 1992; Rosti et al, 1992; Barnett et al, 1993; Motzer et al, 1993; Siegert et al, 1994; Margolin et al, 1996), with a finding of 8% in the largest reported group of 310 patients (Beyer et al, 1996). In this study HDT-associated death occurred in 1 of 31 patients (3%), as a result of acute renal failure, with two other patients requiring temporary haemodialysis. Although this was not a randomized study, these data suggest that adjustment of carboplatin dose to renal function may reduce the early morbidity and mortality associated with HDT without compromising efficacy.

In conclusion, the HDT schedule used in this study appears to be effective in a significant proportion of patients with primary refractory or relapsed (but chemotherapy-sensitive) GCT. The previously noted poor outcome for those with resistant relapse, confirmed in this study, suggests that alternative therapeutic approaches should be explored in these patients.

### REFERENCES

Barnett MJ, Coppin CML, Murray N, Nevill TJ, Reece DE, Klingemann H-G, Shepherd JD, Nantel SH, Sutherland HJ and Phillips GL (1993) High-dose chemotherapy and autologous bone marrow transplantation for patients with poor prognosis nonseminomatous germ cell tumours. *Br J Cancer* **68**: 594–598

Beyer J, Kramar A, Mandanas R, Linkesch W, Greinix A, Droz JP, Pico JL, Diehl A, Bokemeyer C, Schmoll HJ, Nichols CR, Einhorn LH and Siegert W (1996) High-dose chemotherapy as salvage treatment in germ cell

© Cancer Research Campaign 1998

tumors: a multivariate analysis of prognostic variables. *J Clin Oncol* **14**: 2638–2645

Broun ER, Nichols CR, Kneebone P, Williams SD, Loehrer PJ, Einhorn LH and Tricot GJK (1992) Long-term outcome of patients with relapsed and refractory germ cell tumours treated with high-dose chemotherapy and autologous bone marrow rescue. *Ann Intern Med* **117**: 124–128

Calvert AH, Newell DR, Gumbrell LA, O'Reilly S, Burnell M, Boxall FE, Siddik ZH, Judson IR, Gore ME and Wiltshaw E (1989) Carboplatin dosage: prospective evaluation of a simple formula based on renal function. *J Clin Oncol* **7**: 1748–1756

Hitchins RN, Newlands ES, Smith DB, Begent RH, Rustin GJ and Bagshawe KD (1989) Long-term outcome in patients with germ cell tumours treated with POMB/ACE chemotherapy: comparison of commonly used classification systems of good and poor prognosis. *Br J Cancer* **59**: 236–242

Kaplan EL and Meier P (1958) Non-parametric estimation from incomplete observations. *J Am Stat Assoc* **53**: 457–481

Margolin K, Doroshow JH, Ahn C, Hamasaki V, Leong L, Morgan R, Raschko J, Shibata S, Somlo G and Tetef M (1996) Treatment of germ cell cancer with two cycles of high-dose ifosfamide, carboplatin, and etoposide with autologous stem-cell support. *J Clin Oncol* **14**: 2631–2637

Mead GM, Stenning SP, Parkinson MC, Horwich A, Fossa SD, Wilkinson PM, Kaye SB, Newlands ES and Cook PA (1992) The Second Medical Research Council study of prognostic factors in nonseminomatous germ cell tumors. *J Clin Oncol* **10**: 85–94

Mencel PJ, Motzer RJ, Mazumdar M, Vlamis V, Bajorin DF and Bosl GJ (1994) Advanced seminoma: treatment results, survival, and prognostic factors in 142 patients. *J Clin Oncol* **12**: 120–126

Motzer RJ and Bosl GJ (1992) High-dose chemotherapy for resistant germ cell tumors: recent advances and future directions. *J Natl Cancer Inst* **84**: 1703–1709

Motzer RJ, Geller NL, Tan CCY, Herr H, Morse M, Fair W, Sheinfeld J, Sogani P, Russo P and Bosl GJ (1991) Salvage chemotherapy for patients with germ cell tumors. The Memorial Sloan-Kettering Cancer Center experience (1979–1989). *Cancer* **67**: 1305–1310

Motzer RJ, Gulati SC, Tong WP, Menendez Botet C, Lyn P, Mazumdar M, Vlamis V, Lin S and Bosl GJ (1993) Phase I trial with pharmacokinetic analyses of high-dose carboplatin, etoposide, and cyclophosphamide with autologous bone marrow transplantation in patients with refractory germ cell tumors. *Cancer Res* **53**: 3730–3735

Nichols CR, Andersen J, Lazarus HM, Fisher H, Greer J, Stadtmauer EA, Loehrer PJ and Trump DL (1992) High-dose carboplatin and etoposide with autologous bone marrow transplantation in refractory germ cell cancer: an Eastern Cooperative Group protocol. *J Clin Oncol* **10**: 558–563

Peckham MJ (1971) Investigation and staging: general aspects and staging classification. In *The Management of Testicular Tumours*, Peckham MJ. (ed.), pp. 89–101. Edward Arnold: London

Rosti G, Albertazzi L, Salvioni R, Pizzocaro G, Cetto GL, Bassetto MA and Marangolo M (1992) High-dose chemotherapy supported with autologous bone marrow transplantation (ABMT) in germ cell tumors: a phase two study. *Ann Oncol* **3**: 809–812

Siegert W, Beyer J, Strohscheer I, Baurmann H, Oettle H, Zingsem J, Zimmermann R, Bokemeyer C, Schmoll HJ and Huhn D (1994) High-dose treatment with carboplatin, etoposide, and ifosfamide followed by autologous stem-cell transplantation in relapsed or refractory germ cell cancer: a phase I/II study. *J Clin Oncol* **12**: 1223–1231

© Cancer Research Campaign 1998